# Supplementary material for: Clostridia from preterm infants metabolize human milk oligosaccharides to suppress pathobionts and modulate intestinal function in organoids
Source: Nat Microbiol. 2026 Mar 16;11(4):940–59. doi: 10.1038/s41564-026-02297-4 (PMC13056567; doi:10.1038/s41564-026-02297-4)
Supplement: Supplementary file 1 — Supplementary Tables 1–4 and Supplementary Data Figs. 1–4. [file 41564_2026_2297_MOESM1_ESM.pdf]

nature microbiology

Supplementary information

https://doi.org/10.1038/s41564-026-02297-4

Clostridia from preterm infants metabolize human milk oligosaccharides to suppress pathobionts and modulate intestinal function in organoids

In the format provided by the authors and unedited

Table S1. Antibiotics resistance of selected isolates.

Isolate	Species	Vancomycin	Ampicillin	Metronidazole	Penicillin	Meropenem
AM76	*B. breve*	≤0.125	0.0625	1	0.032	≤0.004
BM6-3	*B. breve*	1	0.5	**>8 ***	0.25	>0.5
CS27	*B. bifidum*	2	0.032	4	0.032	0.016
LB1	*B. infantis*	1	0.25	**>8 ***	0.125	0.25
PP1	*B. animalis*	0.5	0.032	2	0.0625	0.032
VK68	*B. breve*	0.5	0.0625	1	0.032	≤0.004
AM1	*C. perfringens*	1	0.016	2	0.0625	0.016
AM37	*C. baratii*	2	0.25	0.5	0.25	0.032
AM226	*C. perfringens*	1	0.0625	0.5	0.0625	0.016
AM248	*C. perfringens*	0.5	0.0625	1	0.0625	0.016
JC25	*C. tertium*	2	1	1	**>1 ***	**0.5 ***
JC26	*C. perfringens*	0.5	0.125	0.5	0.125	0.016
JC29	*C. perfringens*	0.5	0.125	1	0.0625	0.032
JC31	*C. butyricum*	1	0.125	0.5	0.25	0.125
JC33	*C. perfringens*	0.5	0.0625	1	0.125	0.016
JC36	*C. perfringens*	0.5	0.0625	1	0.0625	0.008
JC53	*C. paraputrificum*	0.5	0.0625	0.5	0.0625	0.016
JC66	*C. paraputrificum*	1	0.25	2	0.0625	0.032
TB43	*C. tertium*	2	1	1	**1 ***	**0.25 ***
ATCC 13124	*C. perfringens*	1	0.0625	1	0.0625	0.016
NCTC 9343	*B. fragilis*	>1	>4	0.5	>1	0.125
NCTC 12973	*S. aureus*	1	1	>8	1	0.125
NCTC 12241	*E. coli*	>1	4	>8	>1	0.032

*Denotes resistance to the antibiotic.

Table S2. Number of differentially expressed genes (DEGs) identified in *C. perfringens* pfoA-(AM1) during growth on individual human milk oligosaccharides (HMOs) compared with growth on lactose.

HMO	Number of DEGs	Number upregulated	Number downregulated
DSLNT	326	238	88
LNnT	240	146	94
6’SL	53	31	22

DSLNT, Disialyllacto-N-tetraose; LNnT, Lacto-N-neotetraose; 6’SL, 6'-Sialyllactose

Table S3. Degradation byproducts following growth of *C. perfringens* pfoA$^-$ (AM1) on lactose, Disialyllacto-N-tetraose, and 6'-Sialyllactose.

HMO used for growth	Concentration (g/l)							
	Lactose	6'SL	LNT	LNnT	DSLNT	Fucose	LNB	Sialic acid
Lactose	0.03	n.d.	n.d.	n.d.	n.d.	n.d.	n.d.	n.d.
Lactose	0.02	n.d.	n.d.	n.d.	n.d.	n.d.	n.d.	n.d.
Lactose	0.02	n.d.	n.d.	n.d.	n.d.	n.d.	n.d.	n.d.
DSLNT	0.02	n.d.	0.89	n.d.	n.d.	n.d.	0.136	0.201
DSLNT	0.02	n.d.	0.97	n.d.	n.d.	n.d.	0.143	0.239
DSLNT	0.02	n.d.	0.95	n.d.	n.d.	n.d.	0.153	0.275
6'SL	0.02	n.d.	n.d.	n.d.	n.d.	n.d.	n.d.	n.d.
6'SL	0.02	n.d.	n.d.	n.d.	n.d.	n.d.	n.d.	0.02
6'SL	0.02	n.d.	n.d.	n.d.	n.d.	n.d.	n.d.	0.022

n.d., not detected; 6’SL, 6'-Sialyllactose, LNT, Lacto-N-tetraose; LNnT, Lacto-N-neotetraose; DSLNT, Disialyllacto-N-tetraose; LNB, Lacto-N-biose I

Table S4. Results of per strain PERMANOVA tests comparing the metabolomes of glucose- and HMO-derived cell free supernatant.

Strain	Species	R2	F	P
AM76	*B. breve*	0.4889	3.8265	0.1
LB1	*B. infantis*	0.6921	8.9925	0.1
AM7	*B. longum*	0.3361	2.0246	0.3
AM37	*C. baratii*	0.7465	11.7808	0.1
JC53	*C. paraputrificum*	0.3803	2.4545	0.3
AM1	*C. perfringens*	0.5051	4.0826	0.1
JC36	*C. perfringens*	0.6835	8.638	0.1
JC26	*C. perfringens*	0.4443	3.1981	0.1
JC25	*C. tertium*	0.6218	6.5773	0.1

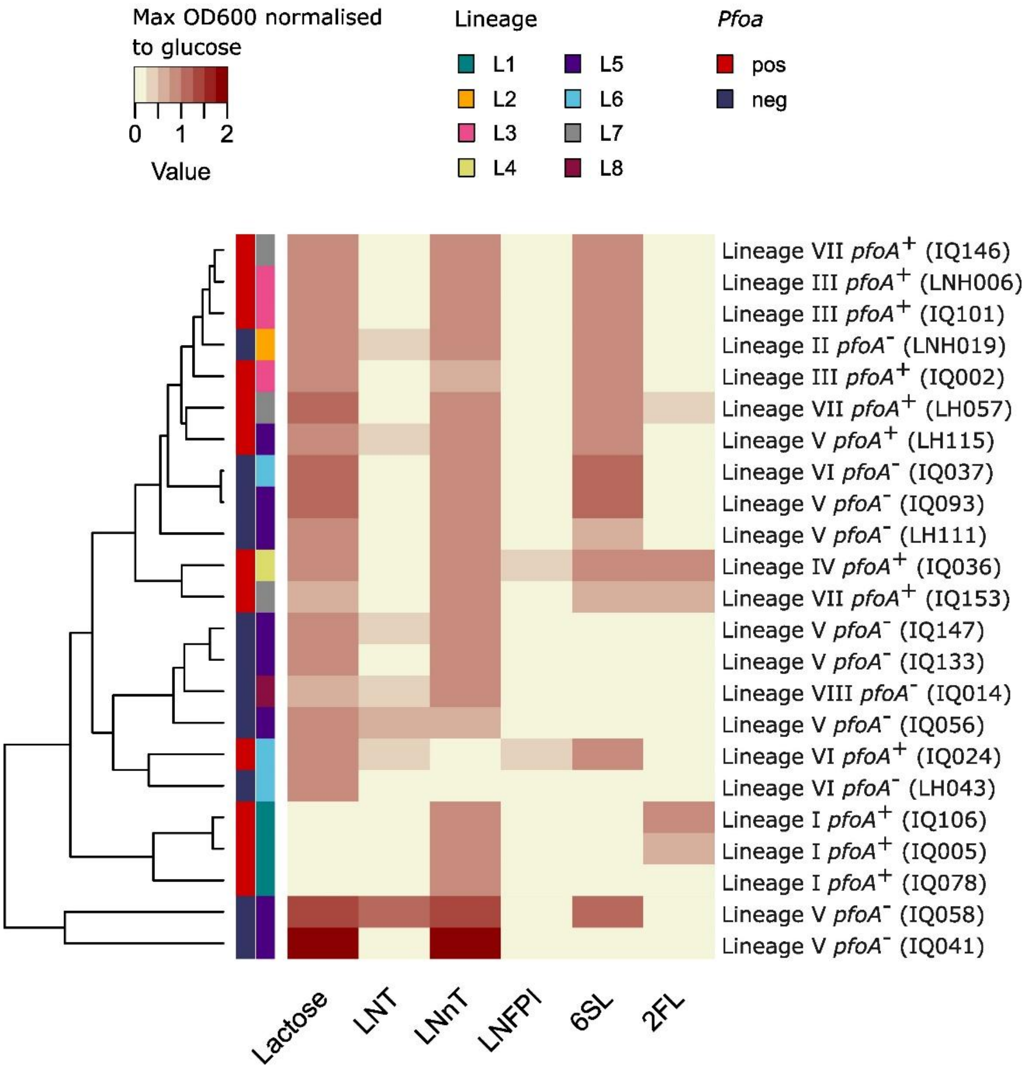

Supplementary Data Figure 1. Growth of *Clostridium perfringens* isolates obtained from Kiu *et al.* (2023)[1] on human milk oligosaccharides (HMOs). Heatmap representing the growth of 23 bacterial isolates on six HMOs and lactose. The values reported represent the maximum OD600 reached normalised to glucose. Growth of these isolates on DSLNT was not tested, due to the limited availability of this HMO.

HMOs, human milk oligosaccharides; LNT, lacto-N-tetraose; LNnT, lacto-N-neotetraose; LNFP I, lacto-N-fucopentaose; 6'-SL, 6'-sialyllactose; 2'FL, 2' fucosyllacotse.

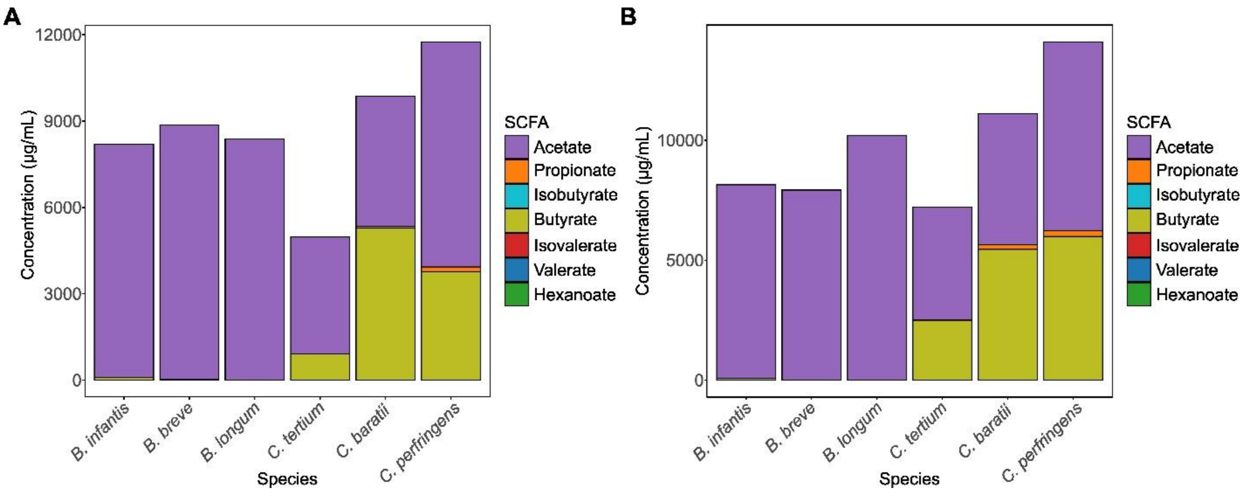

Supplementary Data Figure 2. Total unadjusted concentration of short chain fatty acids in culture supernatants of *Bifidobacterium and Clostridium* spp. grown on glucose (A) and lactose (B). SCFA profiling was performed on culture supernatants harvested following the HMO growth assay shown in Fig 1A. The raw SCFA concentrations for each strain were averaged per species.

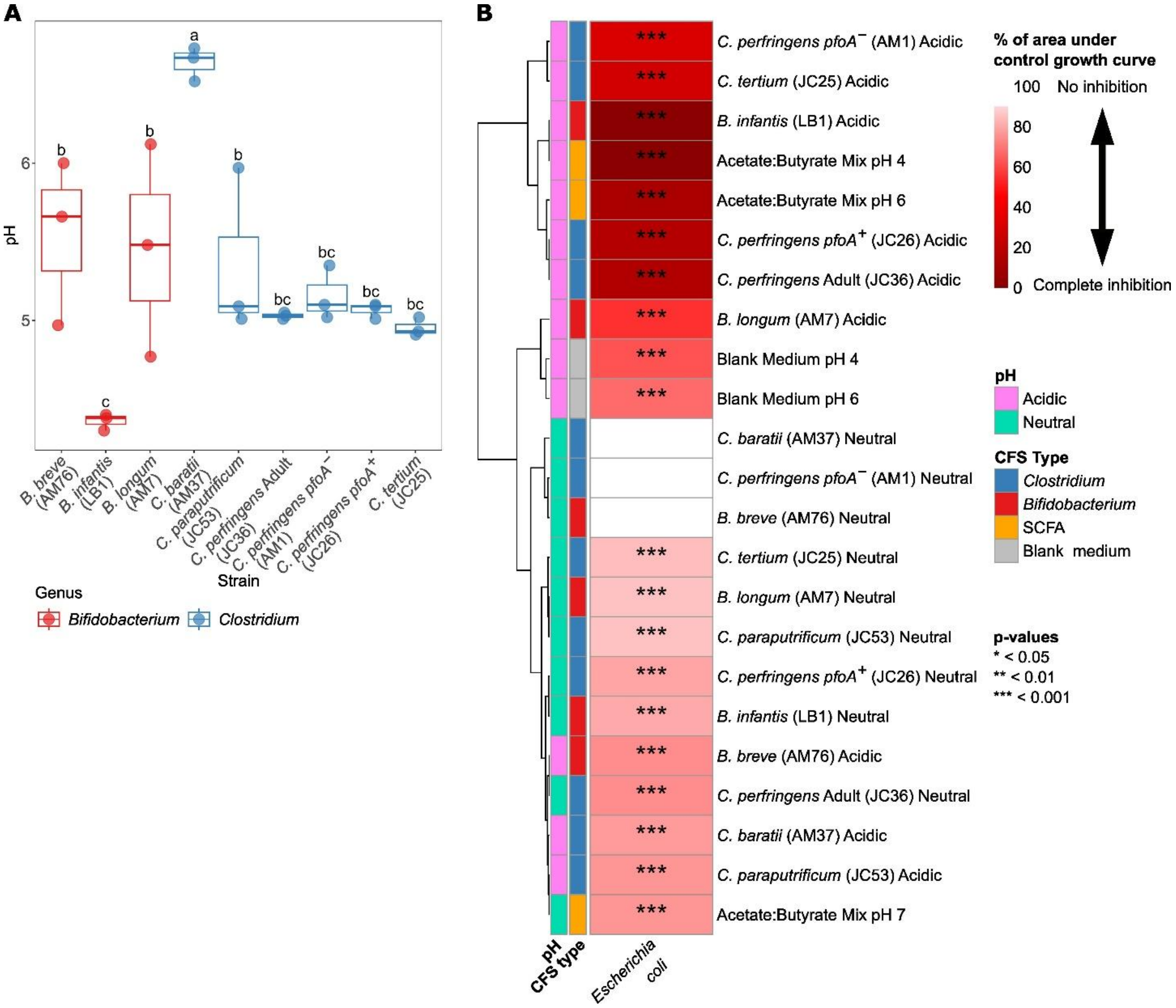

Supplementary Data Figure 3. Effect of pH neutralisation on the antimicrobial activity of all cell free supernatants (CFSs). (**A**) Measured pHs of three batches of each CFS generated (n = 3 per CFS). Conditions with the same letters are not significantly different. Statistical comparisons were performed using an ANOVA, followed by adjustment for multiple comparisons using two-tailed Tukey's HSD method. For all boxplots: centre line, median; box limits, upper and lower quartiles; whiskers, 1.5x interquartile range. (**B**) Comparison of effect of pH neutralisation on activities of all CFSs on *E. coli* growth. Values represent area under the curve (AUC) for growth in media supplemented with CFS, as a percentage of the control AUC. Statistical comparisons were performed using an ANOVA, followed by Dunnett's Test to adjust for multiple comparisons, whereby the growth of each strain following addition of pH7 ZMB1 medium was used as the control.

CFS, cell free supernatants; SCFA, short chain fatty acid.

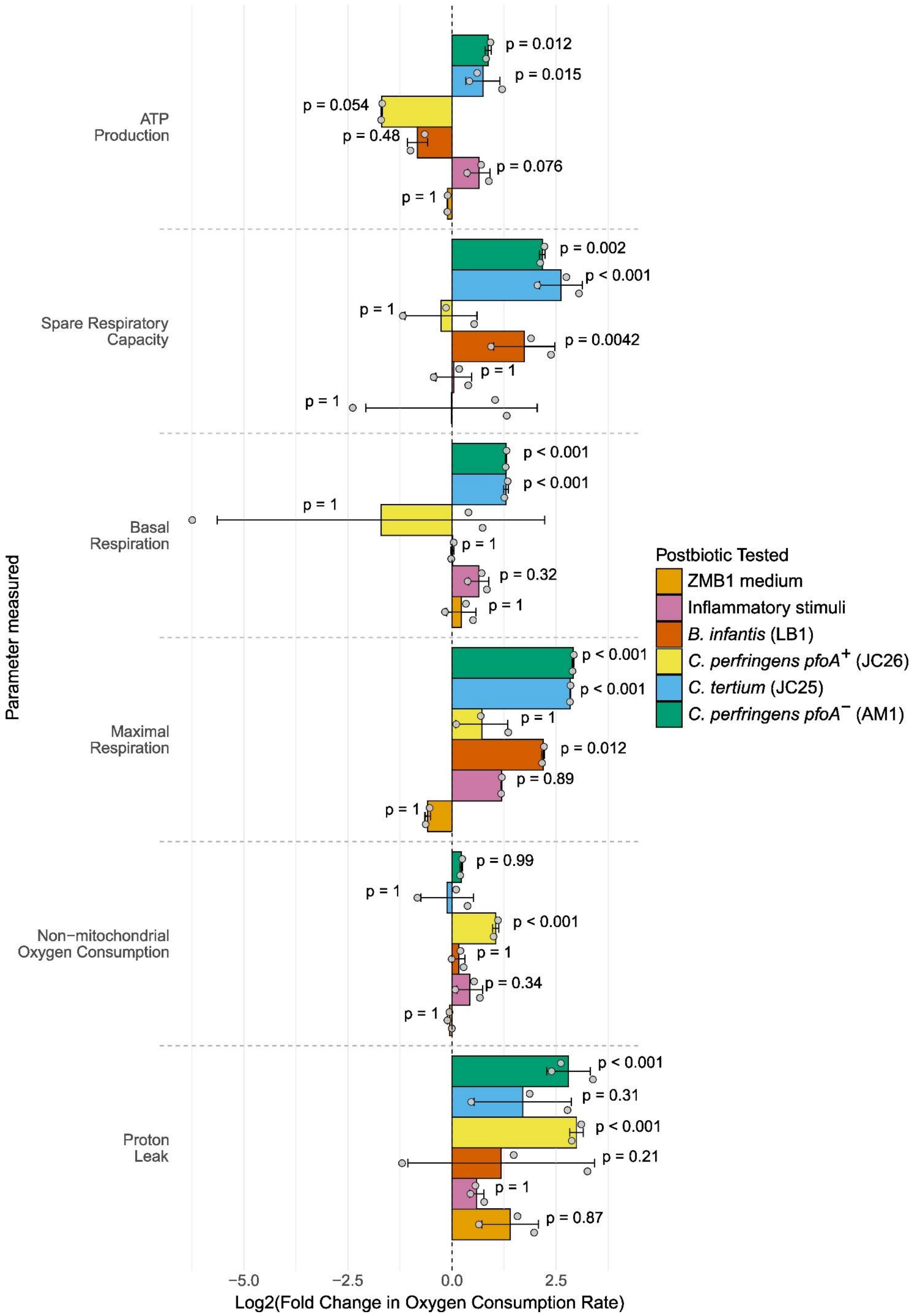

ATP Production
Spare Respiratory Capacity
Basal Respiration
Maximal Respiration
Non−mitochondrial Oxygen Consumption
Proton Leak
Parameter measured
Log2(Fold Change in Oxygen Consumption Rate)
−5.0
−2.5
0.0
2.5
p = 0.012
p = 0.015
p = 0.054
p = 0.48
p = 0.076
p = 1
p = 0.002
p < 0.001
p = 1
p = 0.0042
p = 1
p = 1
p < 0.001
p < 0.001
p = 1
p = 1
p = 0.32
p = 1
p < 0.001
p < 0.001
p = 1
p = 0.012
p = 0.89
p = 1
p = 0.99
p = 1
p < 0.001
p = 1
p = 0.34
p = 1
p < 0.001
p = 0.31
p < 0.001
p = 0.21
p = 1
p = 0.87
Postbiotic Tested
ZMB1 medium
Inflammatory stimuli
B. infantis (LB1)
C. perfringens pfoA+ (JC26)
C. tertium (JC25)
C. perfringens pfoA− (AM1)

Supplementary Data Figure 4. Change in mitochondria bioenergetic parameters for intestinal organoid monolayers exposed to different bacterial cell free supernatants, as well as inflammatory stimuli and ZMB1 medium controls. Data are shown as log2 transformed fold changes in oxygen consumption rate (OCR) compared to 'no treatment' (for each parameter measured, n = 4-6 to calculate fold changes, per CFS tested). P values were calculated for the differences from 'no treatment' using the raw OCR values. Data are presented as mean values +/- standard deviation. Statistical comparisons were performed using an ANOVA, followed by Dunnett's Test to adjust for multiple comparisons, whereby cytokine secretion from "No treatment" was used as the control.
CFS, cell free supernatant.

References

1. Kiu, R., Shaw, A.G., Sim, K., Acuna-Gonzalez, A., Price, C.A., Bedwell, H., Dreger, S.A., Fowler, W.J., Cornwell, E., Pickard, D., et al. (2023). Particular genomic and virulence traits associated with preterm infant-derived toxigenic Clostridium perfringens strains. Nature Microbiology *8*, 1160-1175. 10.1038/s41564-023-01385-z.
