# Supplementary material for: Clostridia from preterm infants metabolize human milk oligosaccharides to suppress pathobionts and modulate intestinal function in organoids
Source: Nat Microbiol. 2026 Mar 16;11(4):940–59. doi: 10.1038/s41564-026-02297-4 (PMC13056567; doi:10.1038/s41564-026-02297-4)
Supplement: Supplementary file 2 — Reporting Summary [file 41564_2026_2297_MOESM2_ESM.pdf]

nature portfolio

Corresponding author(s): Christopher J Stewart

Last updated by author(s): Feb 6, 2026

Reporting Summary

Nature Portfolio wishes to improve the reproducibility of the work that we publish. This form provides structure for consistency and transparency in reporting. For further information on Nature Portfolio policies, see our Editorial Policies and the Editorial Policy Checklist.

Statistics

For all statistical analyses, confirm that the following items are present in the figure legend, table legend, main text, or Methods section.

n/a	Confirmed	
☐	☒	The exact sample size (*n*) for each experimental group/condition, given as a discrete number and unit of measurement
☐	☒	A statement on whether measurements were taken from distinct samples or whether the same sample was measured repeatedly
☐	☒	The statistical test(s) used AND whether they are one- or two-sided<br>*Only common tests should be described solely by name; describe more complex techniques in the Methods section.*
☒	☐	A description of all covariates tested
☐	☒	A description of any assumptions or corrections, such as tests of normality and adjustment for multiple comparisons
☐	☒	A full description of the statistical parameters including central tendency (e.g. means) or other basic estimates (e.g. regression coefficient) AND variation (e.g. standard deviation) or associated estimates of uncertainty (e.g. confidence intervals)
☐	☒	For null hypothesis testing, the test statistic (e.g. *F*, *t*, *r*) with confidence intervals, effect sizes, degrees of freedom and *P* value noted<br>*Give P values as exact values whenever suitable.*
☒	☐	For Bayesian analysis, information on the choice of priors and Markov chain Monte Carlo settings
☒	☐	For hierarchical and complex designs, identification of the appropriate level for tests and full reporting of outcomes
☒	☐	Estimates of effect sizes (e.g. Cohen's *d*, Pearson's *r*), indicating how they were calculated

Our web collection on statistics for biologists contains articles on many of the points above.

Software and code

Policy information about availability of computer code

Data collection

No software were used for data collection.

Data analysis

For analyses using R, v4.4.0
Heatmaps were all visualised with pheatmap v1.0.12
Other plots were visualised with ggplot2 v3.5.0
ANOVA: aov() function in R
Tukey's test: TukeyHSD() function in R, and HSD.test() from agricolae v1.3-7
Dunnett's test: glht() from multcomp v1.4-29
Unpaired T-test: t.test() function in R

Whole genome sequencing:
Assembly: SPAdes
Quality: checkm v1.1.3 and GUNC v1.0.5
Taxonomic assignment: gtdb-tk v2.3.2
Annotation: prokka v1.14
Core gene alignment construction: panaroo v1.2.8
Phylogeny: IQ-TREE v2.0.5
Phylogenetic tree visualisation: iTOL v6.0
Clostridium toxin and colonisation factor gene screen: ABRicate v1.0.1
Genome sizes: sequence-stats v1.0
Mash distance sequence tree: Mashtree v1.2.0

Distance tree visualisation: iTOL v6.0

Screening Clostridium perfringens MAGs for pfoA: BlastN (v2.16.0)

RNAseq of C. perfringens:
Read alignment to genome: Bowtie v2.4.5
Gene level count data: HTSeq v2.0.8
Differential expression analysis: DESeq2 v1.44.0

Proteomics:
Acquired data processing: DIA-NN v1.8
Differential abundance analyses: Limma v3.6.4

Metabolomics data analysis:
PERMANOVA: Vegan v2.6.8
Differential abundance analysis: Limma v3.56.2
Venn diagram of shared metabolites: VennDiagram v1.7.3

CFS microbial activity assay
Area under the curve calculation: caTools v1.18.2

Seahorse mitochondrial stress test assay:
Raw data processing and export: Wave v2.6.3 (Agilent Technologies)

For manuscripts utilizing custom algorithms or software that are central to the research but not yet described in published literature, software must be made available to editors and reviewers. We strongly encourage code deposition in a community repository (e.g. GitHub). See the Nature Portfolio guidelines for submitting code & software for further information.

Data

Policy information about availability of data

All manuscripts must include a data availability statement. This statement should provide the following information, where applicable:

- Accession codes, unique identifiers, or web links for publicly available datasets
- A description of any restrictions on data availability
- For clinical datasets or third party data, please ensure that the statement adheres to our policy

Source data are provided with this paper. All cytokine data from ELISA and multiplex MSD assays are provided in Supplementary tables 5-11. The RNA-seq data have been deposited in the Sequencing Read Archive (SRA) under study accession number PRJNA1214204. The proteomics datasets are deposited in MassIVE under submission ID MSV000096907. Sequencing reads for de novo genomes have been deposited in the European Nucleotide Archive (ENA) under accession number ERP187615.

Research involving human participants, their data, or biological material

Policy information about studies with human participants or human data. See also policy information about sex, gender (identity/presentation), and sexual orientation and race, ethnicity and racism.

Reporting on sex and gender	We do not report on sex and gender.
Reporting on race, ethnicity, or other socially relevant groupings	We do not report on these variables.
Population characteristics	We use intestinal epithelial organoids derived from the resected tissue of a preterm infant.
Recruitment	Preterm infants (born at <32 weeks gestation) were born or transferred to a single tertiary level Neonatal Intensive Care Unit in Newcastle upon Tyne, UK, and participated in the Supporting Enhanced Research in Vulnerable Infants (SERVIS) study (REC10/H0908/39) after written informed parental consent. Parents are approached in the first week of life when the study is explained by a member of the research team. Parents were approached in the first week of life when the study was explained by a member of the research team. Parents were given the option to opt in or out of each specific aspect on a single consent form. Parents of infants who were initially extremely unwell were only approached when they were considered stable by the bedside nurse and medical team. Approaches were by experienced neonatal staff familiar with the studies being described, sample collection, and parental communication. Written signed consent was obtained after the parents have had time to consider the information. Stool samples were regularly collected from nappies/diapers of preterm infants into sterile collection pots by nursing staff. Breast milk samples were collected from residuals from an infant's feeding systems. Samples were initially stored at -20°C before being transferred to -80°C for long term storage. Intestinal tissue samples used to generate organoid cell lines were salvaged following surgical resection. Participants are not compensated for donation.
Ethics oversight	The study protocol was approved by Newcastle Hospitals NHS Foundation Trust (NUTH), NRES Committee North East and N Tyneside 2 10/H0908/39, and the research complies with all relevant ethical regulations.

Note that full information on the approval of the study protocol must also be provided in the manuscript.

Field-specific reporting

Please select the one below that is the best fit for your research. If you are not sure, read the appropriate sections before making your selection.

☒ Life sciences ☐ Behavioural & social sciences ☐ Ecological, evolutionary & environmental sciences

For a reference copy of the document with all sections, see nature.com/documents/nr-reporting-summary-flat.pdf

Life sciences study design

All studies must disclose on these points even when the disclosure is negative.

Sample size

We screened the abilities of 29 bacterial isolates, mostly from preterm infant stool, to grow on six different HMOs, and glucose and lactose. These species were obtained by untargeted cultivation and represent a median of 80% (interquartile range 61%-91%) of all relative microbial abundance observed in preterm infants. Bacterial strains identified as being of interest, based on HMO utilisation profile, were taken forward for systematic experimentation. One organoid cell line was used, with all experiments performed in triplicate, which we deemed important for maintaining a consistent host genetic background for comparing different treatment conditions. For all laboratory experiments, no sample size calculations were performed. Experiments were conducted with n = 3 per condition, based on standard laboratory practice for precision, enabling the identification of and potential correction for outliers.

Data exclusions

For Seahorse mitochondrial stress test assays, organoid trans-epithelial electrical resistances and cytokine assays, outliers were removed from datasets. Outliers were identified by calculating robust/modified z scores for each data point. This method uses the median to calculate median absolute deviation. Any data point with a robust z score greater than +3.5 or less than -3.5 was removed from the dataset. At least two replicates were always retained per group. For this work, we wanted an unbiased, numerical measure for outlier assessment. We ultimately selected robust z scores, as they are more resistant to outlier distortion, due to reliance on median absolute deviation, making this method more appropriate these small datasets.

Replication

All experiments were performed with a minimum of 3 replicates per group, with outliers then removed following the methodology described above.

Randomization

Randomization was not required in this study, as the experimental design involved controlled manipulations of treatment conditions. Responses were measured in a systematic manner, with each treatment applied to a consistent bacterial strain and organoid cell line. This approach ensured reliable comparisons between conditions without the need for random assignment.

Blinding

Blinding was not used in this study, as experimental design involved controlled manipulations of treatment conditions, with each treatment applied to a consistent bacterial strain or organoid cell line.

Reporting for specific materials, systems and methods

We require information from authors about some types of materials, experimental systems and methods used in many studies. Here, indicate whether each material, system or method listed is relevant to your study. If you are not sure if a list item applies to your research, read the appropriate section before selecting a response.

Materials & experimental systems

n/a	Involved in the study	
☒	☐	Antibodies
☐	☒	Eukaryotic cell lines
☒	☐	Palaeontology and archaeology
☒	☐	Animals and other organisms
☒	☐	Clinical data
☒	☐	Dual use research of concern
☒	☐	Plants

Methods

n/a	Involved in the study	
☒	☐	ChIP-seq
☒	☐	Flow cytometry
☒	☐	MRI-based neuroimaging

Eukaryotic cell lines

Policy information about cell lines and Sex and Gender in Research

Cell line source(s)

This study used a preterm intestinal-derived organoid model which was established in the Stewart Lab, Newcastle University using resected surgical ileum tissue that was obtained from the neonatal intensive care unit of the Royal Victoria Infirmary, Newcastle. Informed consent was obtained by parents. The laboratory ID of this organoid cell line is NCL 27 and it was established from a male patient born at 24 weeks gestation, using ileum tissue salvaged from surgery performed on day of life 10 due to the development of necrotising enterocolitis.

Authentication

The cell line was not authenticated.

Mycoplasma contamination

Cell lines were not tested for mycoplasma contamination.

Commonly misidentified lines<br>(See ICLAC register)	No commonly misidentified cell lines were used.

Plants

Seed stocks	*Report on the source of all seed stocks or other plant material used. If applicable, state the seed stock centre and catalogue number. If plant specimens were collected from the field, describe the collection location, date and sampling procedures.*
Novel plant genotypes	*Describe the methods by which all novel plant genotypes were produced. This includes those generated by transgenic approaches, gene editing, chemical/radiation-based mutagenesis and hybridization. For transgenic lines, describe the transformation method, the number of independent lines analyzed and the generation upon which experiments were performed. For gene-edited lines, describe the editor used, the endogenous sequence targeted for editing, the targeting guide RNA sequence (if applicable) and how the editor was applied.*
Authentication	*Describe any authentication procedures for each seed stock used or novel genotype generated. Describe any experiments used to assess the effect of a mutation and, where applicable, how potential secondary effects (e.g. second site T-DNA insertions, mosiacism, off-target gene editing) were examined.*
